# Application of Biomass Corrosion Inhibitors in Metal Corrosion Control: A Review

**DOI:** 10.3390/molecules28062832

**Published:** 2023-03-21

**Authors:** Qihui Wang, Ruozhou Wang, Qi Zhang, Chongkang Zhao, Xing Zhou, Huahao Zheng, Rui Zhang, Yi Sun, Zhitao Yan

**Affiliations:** 1 School of Civil Engineering and Architecture, Chongqing University of Science and Technology, Chongqing 401331, China; 2 Chongqing Key Laboratory of Energy Engineering Mechanics & Disaster Prevention and Mitigation, Chongqing 401331, China

**Keywords:** corrosion inhibitor, inhibition mechanism, biomass, preparation method

## Abstract

Corrosion is the process of damaging materials, and corrosion of metallic materials frequently results in serious consequences. The addition of corrosion inhibitors is the most effective means of preventing metal corrosion. Until now, researchers have made unremitting efforts in the research of high-efficiency green corrosion inhibitors, and research on biomass corrosion inhibitors in a class of environmentally friendly corrosion inhibitors is currently quite promising. This work presents the classification of green biomass corrosion inhibitors in detail, including plant-based corrosion inhibitors, amino acid corrosion inhibitors, and biosurfactant corrosion inhibitors, based on the advantages of easy preparation, environmental friendliness, high corrosion inhibition efficiency, and a wide application range of biomass corrosion inhibitors. This work also introduces the preparation methods of biomass corrosion inhibitors, including hydrolysis, enzymatic digestion, the heating reflux method, and microwave extraction. In addition, the corrosion inhibition mechanisms of green biomass corrosion inhibitors, including physical adsorption, chemisorption, and film-forming adsorption, and evaluation methods of biomass corrosion inhibitors are also explicitly described. This study provides valuable insights into the development of green corrosion inhibitors.

## 1. Introduction

Metal materials are extensively utilized in the construction, petroleum, metallurgical, and aerospace industries as a result of their excellent physical and chemical properties, and high ductility [1]. Metals are highly active, resulting in an oxide film on the metal surface under the action of an aerobic environment. Metal corrosion is the process by which the action of surrounding media damages metal materials with the ingress of corrosion ions. Metal corrosion is a spontaneous chemical reaction that can be divided into chemical corrosion and electrochemical corrosion, according to the reaction mechanism [2]. Whether chemical corrosion or electrochemical corrosion occurs, metallic materials and chemical substances are subject to redox reactions. The oxidation-reduction reactions of metals become stronger over time, and eventually metals lose their original capacity.

The damage to metal materials caused by metal corrosion is controlled and preventable compared with the damage caused by other natural disasters [3]. According to the available statistics, contemporary industrial technology is capable of efficiently reducing corrosion-related dangers and economic losses by 20 to 30% if proper corrosion-prevention techniques are utilized. Therefore, metal corrosion prevention and control have significant value. Currently, there are numerous methods for preventing and controlling metal corrosion, including the use of high-performance corrosion-resistant metals, protective coatings covering the metal surface, electrochemical technology to prevent corrosion, oxidation and phosphorylation of metal surfaces, and the addition of corrosion inhibitors [4]. The addition of corrosion inhibitors is one of the necessary corrosion prevention measures. Therefore, this work mainly explains the application of corrosion inhibitors to metal corrosion protection.

Baldwin explained how corrosion inhibitors prevent corrosion in his first patent application for corrosion inhibitors in 1860. Inorganic corrosion inhibitors are inorganic compounds that play a significant role in corrosion inhibition and mainly include nitrite, chromate, silicate, molybdate, polyphosphate, tungstate, zinc salt, etc. [5]. In recent years, researchers have made further developments in the research of corrosion inhibitors by using synthetic substances as corrosion inhibitors to produce organic corrosion inhibitors with excellent corrosion inhibition properties. After continuous research, Ahamad [6] proposed that we can now detect the inhibition process of corrosion on metal surfaces using weight-loss methods, electrochemical methods, molecular dynamics simulations, quantum chemical calculations, etc. Patchaiah [7] demonstrated that Artemisia annua extract in hydrochloric acid solution provides effective corrosion inhibition to prevent carbon steel corrosion. This study provides an experimental case for the development of green corrosion inhibitors. Many researchers have extensively researched environmental corrosion inhibitors and conducted numerous reviews to summarize the most helpful information on corrosion inhibition efficiency.

This study analyses the behavior and characteristics of different corrosion inhibitors affecting metal corrosion, summarizes the mechanisms that inhibit corrosion, and reviews biomass corrosion inhibitors based on extensive literature studies. This research demonstrates a theoretical and practical foundation for future research on the corrosion of metals in corrosive environments.

## 2. Classification of Biomass Corrosion Inhibitors

**Figure 1:**
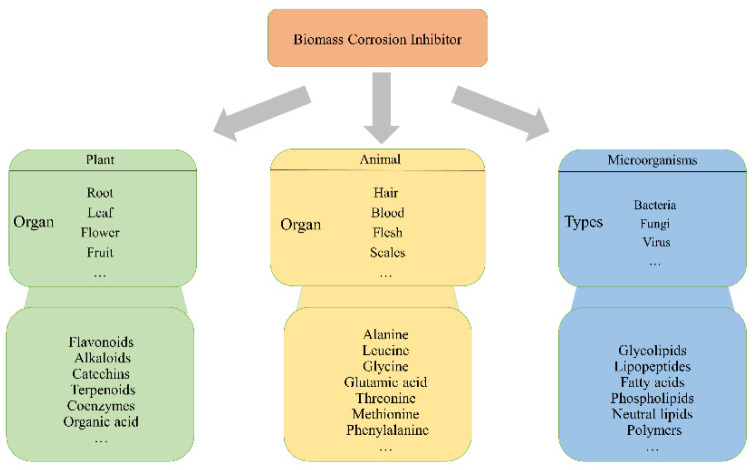
Schematic diagram of the source of biomass corrosion inhibitors.

Due to the high efficiency and convenience of corrosion inhibitors, they have been extensively used as an effective means of metal prevention. Researchers have discovered many chemicals with diverse corrosion inhibition mechanisms that serve as corrosion inhibitors in various environments. Biomass corrosion inhibitors are available from numerous sources, including plants, animals, and microorganisms, as illustrated in Figure 1. The three types of corrosion inhibitors are scattered extensively in nature. Corrosion inhibitors extracted from fruits, leaves, stems, flowers, and roots of plants have many advantages, such as low cost, eco-friendliness, abundant sources, and low toxicity [8]. Amino-acid-based corrosion inhibitors can be obtained from protein hydrolysis products. Because of their merits of being green, inexpensive, and widely available, they have attracted much attention from researchers and have achieved remarkable results in the corrosion prevention of metals [9]. Biosurfactants obtained from the metabolic process of microorganisms are non-toxic, biodegradable, ecologically safe, and highly surface active, have research and development implications in several industries, and have been applied to prevent the corrosion of metals [10].

### 2.1. Corrosion Inhibitors Extracted from Plants

The disadvantages of inorganic corrosion inhibitors include high costs, environmental toxicity, inability to degrade, and easy environmental pollution. Corrosion inhibitors extracted from green plants are widely studied as an effective alternative to traditional toxicity inhibitors [11]. Plant extracts usually contain amino acids, proteins, polysaccharides, and vitamins. A plant is composed of water-soluble metabolites such as organic acids, quinones, phenolic compounds, flavonoids, alkaloids, catechins, terpenoids, and coenzymes, which are friendly to the environment [12]. Therefore, plant extracts are utilized as organic corrosion inhibitors.

The active extract molecules in plant-based corrosion inhibitors contain alkaloids, sugar tannins, amino acids, etc. Alkaloids are organic alkaline substances, most of which contain heteroatoms and form cyclic compounds. Sugars have polyhydroxy aldehydes and polyhydroxy ketones, which can be hydrolyzed to produce organic compounds of polyhydroxy aldehydes or polyhydroxy ketones, and tannins are natural polyphenolic compounds. Amino acids are organic compounds that contain basic amino and acidic carboxyl groups. These molecules contain unpaired electron elements such as O, N, S, and P compounds, and various chemical substances containing polar groups. These organic substances undergo adsorption on the metal surface and cover the metal surface, thus preventing corrosion of the metal.

There have been many advances in corrosion inhibitors derived from plant extracts in recent years. According to a literature survey, many studies have reported using extracts of roots, peel, stems and leaves, fruits, and seeds of plants as corrosion inhibitors. Rocha [13] studied a corrosion inhibitor capable of slowing the corrosion of carbon steel in acid solutions, the main component of which was extracted from fruit peels, with the characteristics of low cost and environmental friendliness. Using surface analysis and electrochemical impedance spectroscopy, the researchers analyzed the anti-corrosion effectiveness of the corrosion inhibitor on carbon steel in an acidic environment. It was shown that the peel extract could effectively inhibit the corrosion of carbon steel in 1 mol/L HCl solution, and the corrosion efficiency increased with the extract concentration, with the highest corrosion inhibition efficiency reaching 96%. By utilizing the weight loss method and EIS, Ji [14] evaluated how banana peel extract inhibited and adsorbed mild steel corrosion in 1 mol/L hydrochloric acid solution. In this study, Tafel polarization curves showed that banana peel extract was effective in reducing the dissolution of mild steel. The AFM images showed that banana peel extract significantly reduced the surface roughness of mild steel. Moreover, banana peel extracts were analyzed using infrared spectroscopy. The extract’s basic structure was obtained to lay the foundation for the later study of the corrosion inhibition mechanism. The adsorption model of banana peel extract was investigated, and the results showed that the Langmuir isotherm model was the most suitable.

Krishnaveni [15] studied the adsorption and corrosion inhibition of carbon steel in sulfuric acid solution by electrochemical methods and applied adsorption theory to process the experimental data. It was found that safranin extract exhibited good corrosion inhibition in hydrochloric acid medium. The potentiodynamic polarization curves showed that flavopiridol inhibited both cathodic and anodic reactions. Rocha et al. [16] demonstrated that white grape pomace extract inhibited the corrosion of carbon steel in a 1 mol/L hydrochloric acid solution. The inhibitory potency of 1 mol/L hydrochloric acid on carbon steel increased with increasing concentrations of concentrated grape pomace extract and varied with temperature. The inhibition was achieved by the adsorption of chemicals from grape pomace extract on the surface of the steel. Flavonoids are viable candidates for elucidating the preservative properties of grape pomace extracts. The dissolved concentration of carbon steel decreased in the presence of grape pomace extract. Scanning electron microscopy showed that the surface of the carbon steel remained smooth after the addition of grape pomace extract, which may have been due to the formation of phenolic compound adsorption films with electrostatic properties.

The extraction of corrosion inhibitors from plants is in line with the concept of green chemistry. Plant extract corrosion inhibitors can reduce the harm inflicted on the human body and environment, which meets the development needs of today’s society. Research on plant extract corrosion inhibitors should start from the extraction method, further study their corrosion inhibition effect, grasp their development direction, form a research system, and promote the development of plant extract corrosion inhibitors.

### 2.2. Amino-Acid-Based Corrosion Inhibitors Extracted from Protein

**Figure 2:**
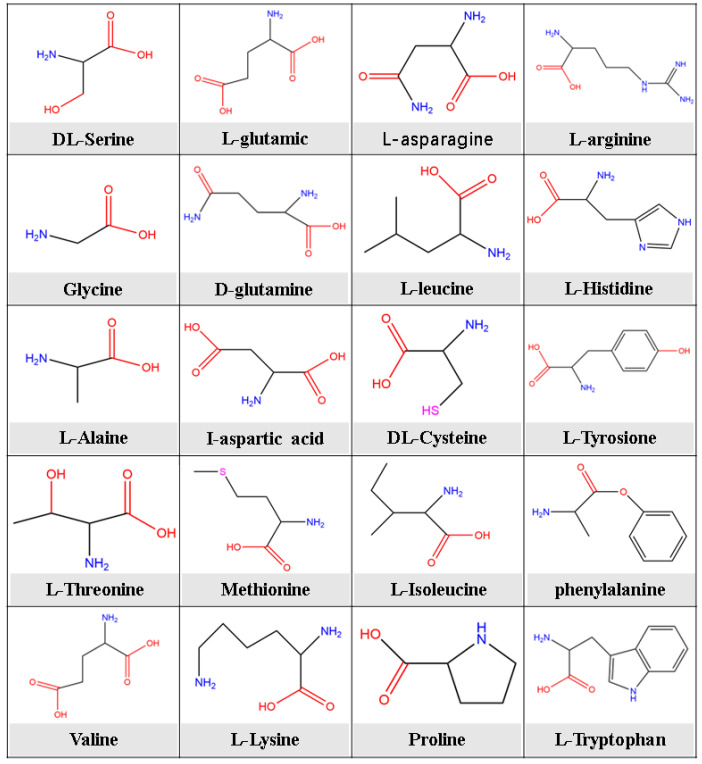
Molecular structure of common amino acids that can be extracted from proteins.

The main substance in an organism is protein, which usually consists of more than 20 amino acids, as shown in Figure 2. Amino acids are the main components of animal proteins and widely used natural compounds that contain amino and carboxyl groups in their molecular structure [9]. Amino acids are classified as acidic, neutral, and basic amino acids. When the number of carboxyl groups in an amino acid is more than that of amino groups, they are acidic, and when the opposite is true they are basic. Meanwhile, when the number of amino and carboxyl groups is close to each other, they can be considered neutral. This particular structure indicates tremendous potential for their application as corrosion inhibitors. Amino acids have been systematically researched as corrosion inhibitors in corrosive media to control the inhibition of corrosion reactions of metals [17].

Lima [18] investigated the corrosion inhibition of mild steel by glycine (Gly) in 0.5 mol/L H_2_SO_4_ and analyzed the total protein content of several isolated proteins using two distinct methods. The observations revealed that Gly effectively inhibited corrosion in H_2_SO_4_ at a concentration of 0.5 mol/L, and this efficacy improved with the concentration of the corrosion inhibitor. The data suggested that the chemical was a corrosion inhibitor of mixed type. Zerfaoui [19] investigated five amino acids, including aspartic acid (Asp), arginine (Arg), glycine (Gly), leucine (Leu), and methionine (Met). The research confirmed the effectiveness of these five amino acids in inhibiting corrosion primarily through weight loss, polarization, and EIS measurements. The experiment demonstrated that each of the five amino acids exerted a corrosion-inhibiting effect on pure iron in a 2 × 10^−2^ mol/L citric acid solution. The results of the electrochemical experiments ranked their corrosion inhibition efficiency as Gly < Leu < Asp < Arg < Met, and the maximum corrosion inhibition effectiveness was 96%, with Met being the most effective.

**Figure 3:**
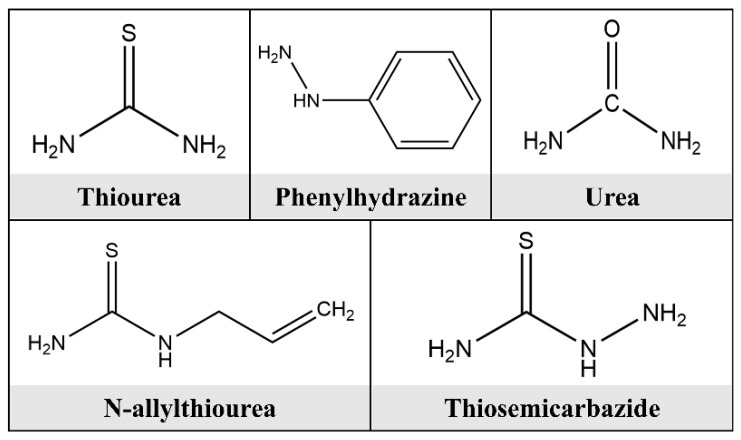
Chemical molecular structures of thiourea, phenylhydrazine, urea, N-allylthiourea, and thiosemicarbazide.

The use of amino acids as corrosion inhibitors is mainly attributed to the excellent adsorption properties of electronic elements or groups. Therefore, amino acids mostly have promising corrosion inhibition effects. Aal [20] experimentally discovered that organic compounds containing sulfur and nitrogen atoms demonstrated superior corrosion inhibition properties. Research has illustrated that cysteine has a sulfhydryl group. The hydrogen ions on the sulfhydryl group can ionize in water, and its negative ions combine with metal ions to form a very complex protective film. This tight protective layer prevents the metal from being further eroded. Haleem [21] discovered that organic compounds containing sulfur or nitrogen atoms and their derivatives could perform well in corrosion inhibition. It was demonstrated that phenylhydrazine, urea, thiourea, N-allylthiourea, and thiosemicarbazide have excellent corrosion inhibition efficiency for aluminum in HCl solution, as shown in Figure 3. Bobina [22] investigated the corrosion inhibition properties of L-histidine on carbon steel in weak acid solutions. The findings of the experiment demonstrated that the adsorption of amino acids on the metal surface followed the Langmuir isotherm, and the Gibbs energy value revealed the electrostatic interaction between charged molecules and carbon steel. L-histidine can physically adsorb to the surface of carbon steel to form an adsorption film, which provides effective protection of carbon steel.

Amino acids contain heteroatoms such as N and O. As a result of these heteroatoms, lone pairs of electrons form coordination complexes with the metal, which retards corrosion of the metal [23]. Previous reports have meticulously elucidated that amino acids are nontoxic and biodegradable and have other environmentally friendly properties. Therefore, amino acids extracted from proteins and employed as corrosion inhibitors for metals have become a hot research topic in recent years.

### 2.3. Extraction of Surfactants from Microorganisms as Corrosion Inhibitors

Biosurfactants refer to substances with surface activity produced by microorganisms through metabolism. The main advantage of biosurfactants is that they are environmentally friendly and can be both biodegraded and used as biodegradation aids to promote the degradation of pollutants. Biosurfactants have convenient access owing to their metabolic production by microorganisms, solid regenerative capacity, low cost, and renewability [24]. Biosurfactants are incredibly effective in inhibiting the corrosion of metals and have excellent environmental adaptability in highly alkaline, chloride-salt environments and acidic conditions.

Zin [25] investigated the effect of rhamnolipid biosurfactant complexes on the corrosion and passivation of freshly cut aluminum–copper–magnesium aluminum alloy surfaces. It was demonstrated that the rhamnolipid biosurfactant effectively inhibited the corrosion of the alloy in synthetic acidic rainwater. The inhibition efficiency became more robust with increasing biosurfactant concentration. Parthipan [26] investigated the corrosion inhibition of carbon steel using a rhamnolipid biosurfactant produced by the bacterial strain Pseudomonas mousseline F01 as an eco-friendly biosurfactant. The results showed that Pseudomonas mosselii F01 formed glycolipid-type surfactants. The adsorption of surfactant molecules on the surface of carbon steel is due to the higher energy of interaction between biosurfactant molecules and the surface of carbon steel than between water and carbon steel. Biosurfactants have corrosion inhibitor and biocide properties and, therefore, can serve as promising biosurfactants.

Shubina et al. [27] evaluated the corrosion inhibition efficiency of reinforcement in a simulated concrete pore solution of lipopeptide secreted by gram-negative bacteria. The electrochemical measurements showed that the lipopeptide changed the chemical conditions of the corrosion products by forming an ohmic resistance region between the surface of the carbon steel and the electrolyte, blocking the anode and cathode reaction sites. The regions moderated the galvanic reactions and the loss of electrons from the metal substrate, leading to a slowdown in the corrosion process. Mobin et al. [28] investigated the corrosion inhibition efficiency of ethane-1,2-diyl bis (N,N-dimethyl-N-hexadecylam-moniumacetoxy) dichloride at different temperatures. The 16-E2-16 organism was synthesized and described using standard electrochemical measurements, microscopic observations, and spectrophotometric analysis to indicate inhibition in simulated concrete pore solutions. Electrochemical measurements and SEM revealed the nature of a protective inhibitory film, a hybrid inhibitor of film-formation biomolecules.

In summary, biosurfactants are remarkably effective in inhibiting metal corrosion. Moreover, biosurfactants are more in line with the global low-carbon and green development strategy because of their environmentally friendly synthesis route, low toxicity, rich structure, and superior performance. Biosurfactants, as a new type of green corrosion inhibitor, have become a hot spot in the field of corrosion inhibitor research. Table 1 shows a comparison of the corrosion inhibition performance of corrosion inhibitors extracted from plants, animals, and microorganisms. In Equation (1), $R_{\mathrm{ct}}$ and $R_{\mathrm{ct}}^{0}$ represent the transfer resistance in solution and the blank solution, respectively. In Equation (2), $i_{\mathrm{corr}}^{0}$ and $i_{\mathrm{corr}}$ represent the corrosion current density in solution and the blank solution, respectively. The η is the corrosion inhibition efficiency.

(1) $$\eta_{R}=\frac{R_{\mathrm{ct}}-R_{\mathrm{ct}}^{0}}{R_{\mathrm{ct}}}\times 100\%$$

(2) $$\eta_{P}=\frac{i_{\mathrm{corr}}^{0}-i_{\mathrm{corr}}}{i_{\mathrm{corr}}^{0}}\times 100\%$$

## 3. Preparation Method of Biomass Corrosion Inhibitor

There are various extraction methods for corrosion inhibitors, and the commonly used extraction methods include the immersion method, heating reflux method, enzymatic digestion method, and microwave extraction method. Among them, the immersion method is simple and convenient to operate, but, at the same time, there are problems such as low efficiency and low extraction rate. The heating reflux method and the Soxhlet extraction method have similar extraction principles and long extraction times, but the Soxhlet extraction method utilizes less solvent and has a higher extraction efficiency. The enzyme activity in the enzymatic digestion method is related to the temperature, pH, and concentration of reactants, which is demanding for the extraction environment and has a restricted scope of application. Furthermore, if the extracted corrosion inhibitor is a liquid, the problem of the difference between the extracted solvent and the solvent in the used environment must be considered to avoid the influence of impurity factors on the corrosion inhibition effect. Liu et al. [34] studied Platanus acerifolia leaf (PAL) extracts using different extraction methods, and the corrosion inhibitors obtained corrosion inhibition effects differently. Therefore, the selection of the appropriate extraction method is essential.

### 3.1. Immersion Method

The immersion method involves immersing the crushed material in the extraction solvent for a certain period, causing the active substance to dissolve and subsequently be extracted. The suspended solids are separated by filtration. The immersion method can extract the most active substances without destroying their chemical properties. The extracts are obtained by soaking in solvents, which are ethanol, hydrochloric acid, or sulfuric acid, for a while and then filtered and can be extracted several times to combine the extracts. Pereira et al. [51] used the immersion method to extract corrosion inhibitors from garlic peel, which was immersed in distilled water at a specific temperature for some time, filtered, and cold-dried to obtain the extract. The sulfide in the corrosion inhibitor composition had a crucial impact on the corrosion inhibition process, and the corrosion inhibitor for carbon steel in 1 mol/L HCl showed an increase in corrosion inhibition efficacy with rising temperature. Jyothi et al. [52] examined the corrosion inhibition performance of methanol extract of Luffa aegyptiaca leaves (MLA) in HCl solution on mild steel. The findings demonstrated that, as the concentration and ambient temperature increased, the ability of MLA to suppress corrosion declined. This corrosion inhibitor is a hybrid that can inhibit both the anodic and cathodic reactions of metals. In addition, halogen ions were found to promote their corrosion inhibition properties, indicating a synergistic corrosion inhibition effect of halogen ions on lucerne leaf extract.

### 3.2. Enzymatic Digestion

Enzymatic methods release active ingredients by hydrolyzing or disrupting the cell wall structure. Therefore, adding enzymes can significantly reduce the extraction time, promote the solubilization of active ingredients in plants, and increase the yield. This method also has the advantages of mild extraction conditions, safe operation, and low pollution. Suitable active enzymes (pectinases, proteases, etc.) are used to hydrolyze specific substances contained in the plant. A suitable environment allows the enzymes to remain active, producing a higher volume of plant extracts. In the enzymatic digestion method, it should be considered whether the added enzymes can affect the corrosion of the metal. Abou-Elseoud et al. [53] used pectin extracted from sugar beet pulp by enzymatic digestion as a corrosion inhibitor. Beet pulp was extracted by enzymatic and acid hydrolysis methods and compared. The findings demonstrated that the pectin extracted had higher corrosion inhibition efficiency than the acid hydrolysis method. The increase in temperature of the solution leads to the desorption of water from the steel surface and an increase in the adsorption of pectin, and the adsorption of pectin molecules prevents the generation of corrosion on the metal surface. Yadav et al. [54] found that natural carbohydrate polymers found in lignocellulosic biomass are biopolymers that can be used in various industrial applications. They can be used as an excellent corrosion inhibitor, and enzymatic digestion is one of the most critical methods.

### 3.3. Heating Reflux Method

The heating reflux method uses an apparatus such as a Soxhlet extractor to obtain the desired plant extract by heating under reflux, filtration, and rotary evaporation using organic solvent as the extraction medium. Haldhar et al. [55] used the heating reflux method to extract the active ingredients from citrus leaves using 250 mL of distilled water as solvent under controlled temperature conditions. Moreover, weight loss, Tafel curve, and electrochemical impedance spectroscopy were used to determine the corrosion prevention efficiency of bamboo leaf extract on mild steel in 0.5 mol/L H_2_SO_4_ solution. The surface roughness of the steel was investigated using SEM, and it was visually observed that citrus leaf extract had an excellent corrosion inhibition effect. Soltani et al. [56] prepared silymarin extract using the heating under reflux method and analyzed the adsorption characteristics of the extract on the surface of steel using quantum chemical calculations. The extracts inhibited the corrosion of stainless steel by adsorption on its surface, and the corrosion inhibition effect was 96%.

### 3.4. Microwave Extraction Method

Microwave-assisted extraction refers to using electromagnetic radiation from microwaves to cause rapid breakage of plant cells, allowing the effective substances in them to flow out, effectively increasing the yield. Microwaves have the advantages of very strong penetration, accelerated molecular vibration, rapid temperature rise, etc., because microwave radiation accelerates the speed of chemical reactions, thus shortening the time required for the reaction and effectively increasing the yield. Suedile et al. [57] prepared the active ingredients of garlic vine extract using the microwave technique and studied the behavior of the extract on Zn metal with the help of electrochemical impedance spectroscopy and polarization curves. The results indicated that the corrosion inhibition efficiency of garlic vine extract on zinc reached 90%, and the principle of action was mainly attributed to the adsorption of organic components in the extract through heteroatoms and π-bonds, which produced a covering effect and promoted metal passivation, reducing the area of metal corrosion. Using a microwave method, Singh et al. [58] synthesized a bisphenol formaldehyde polymer containing piperazine to examine its corrosion inhibition on mild steel in 1 mol/L HCl. The pH was adjusted with sodium hydroxide accordingly. Then, the mixture was kept in the microwave with continuous stirring at a fixed temperature, and piperazine was added to the mixture. The reactants were cooled and filtered to obtain the extract. A good corrosion inhibition effect was obtained in various corrosion inhibition characterizations.

## 4. Inhibition Mechanism of Metal Corrosion by Biomass Corrosion Inhibitors

This work describes the different mechanisms of metal corrosion inhibition by biomass corrosion inhibitors in recent studies. Furthermore, the work emphasizes the specific corrosive medium under study. The inhibitory behavior of the different active substances contained in biomass corrosion inhibitors varies considerably depending on environmental factors. The corrosion of metals occurs under certain conditions. The corrosion inhibition mechanisms for different metals can be divided into three categories: physical adsorption, chemisorption, and film-forming adsorption.

### 4.1. Physical Adsorption

Physical adsorption utilizes van der Waals forces, mainly between the corrosion inhibitor molecules and the metal surface. Since van der Waals forces are weak, the adsorption process requires little heat, and the adsorption process is slow, so physical adsorption is strongly reversible. The adsorption film formation is not solid. In the organic corrosion inhibitor adsorption process, physical adsorption generally occurs in front of chemical adsorption and is the preparatory stage of chemical adsorption [59].

Using Taxus baccata extracts in methylene dichloride (MDE), ethyl acetate (EAE), and butyraldehyde (BE), Hanin et al. [60] investigated the mechanism of corrosion inhibition of carbon steel in 1 mol/L HCl solution. The results demonstrated that extracts of Taxus baccata were mixed-type inhibitors in the polarization curve. The adsorption of its corrosion inhibitor molecules on the carbon steel surface was attributed to the lower energy of the interaction between the corrosion inhibitor molecules and the carbon steel surface than between the water molecules and the carbon steel. The mechanism of corrosion inhibition of plant-based corrosion inhibitors formed by different polyphenol extracts of Taxus baccata can thus be ascribed to the interaction between the protonated inhibitor molecules and the cations of charged metals, indicating the presence of physical adsorption.

In an HCl solution, Gerengi et al. [61] examined the corrosion inhibition effect and mechanism of Schinopsis lorentzii extract on low carbon steel. Temkin adsorption isotherms confirmed that physical-type monolayer adsorption occurred between molecules and metals, and that the resulting electrostatic interactions inhibited the corrosion of metals. The functional group of this molecule has O–H, C=C, C–H, and C–O bonds. The oxygen atom and the aromatic ring of the active group are typical plant-based corrosion inhibitors. In acidic media, the nonbonded electrons of oxygen are protonated. As a result of electrostatic interactions, molecules of protonated components are adsorbed. FTIR has confirmed the presence of aromatic and heteroaromatic groups in Schinopsis lorentzii extract, indicating that its protective layer on the metal surface is formed by the electrostatic interaction between corrosion inhibitor molecules and iron ions during the exchange of the solution with the inhibitor.

### 4.2. Chemisorption

During chemical adsorption, inhibitors often form complexes on a metal surface through the transfer of electrons and the formation of coordinated covalent bonds. Since the corrosion inhibitor molecule contains elements with non-covalent electron pairs, such as N, O, S and P, these electrons combine with the metal ligands to form a strong chemisorbed layer. These electrons interact with the solution’s oxygen ions and repel the cations’ proximity due to the electrostatic force. Meanwhile, these electrons can enter into the empty orbitals of the metal elements, forming coordination bonds that adsorb to the metal surface, thus protecting the metal [62]. Chemical adsorption generally occurs at the completion stage of corrosion inhibitor adsorption. The adsorption process requires more energy, strong adsorption, and higher corrosion inhibition efficiency.

Most chemisorption corrosion inhibitors contain hydrophilic and hydrophobic groups, and hydrophilic groups can adsorb on to the surface of the metal. Meanwhile, the hydrophobic group presents a backward state, whereby the group covers the active center of the metal. It reduces the contact of the metal with water and with dissolved oxygen, thus providing a corrosion inhibition effect. Zhang et al. [63] fermented Pseudomonas aeruginosa and Burkholderia cepacia to obtain the secondary metabolite rhamnolipid. The study showed that rhamnolipid was an anodic-based corrosion inhibitor with more than 90% efficiency for X65 steel at 40 mg/L. Rhamnolipid was chemisorbed on the steel surface with an optimal adsorption time of 2 h. The surface analysis results showed that the adsorption mechanism of rhamnolipid is mainly a rhamnopyranose ring and C=O groups, which provide electrons to bind iron atoms through the hollow orbitals.

Dong et al. [64] investigated the corrosion inhibition mechanism of exopolysaccharide (EPS) extracted from thermophilic sulfate-reducing bacteria (SRB) on Q235 carbon steel under H_2_SO_4_. Corrosion began with the production of Fe^2+^ by anodic oxidation, and the reaction was as follows:

(3) $$\mathrm{Fe}\to {\mathrm{Fe}}^{2+}+2e^{-}$$

(4) $$\mathrm{Fe}\to {\mathrm{Fe}}^{3+}+3e^{-}$$

(5) $$\mathrm{Fe}+2{\mathrm{Fe}}^{3+}\to 3{\mathrm{Fe}}^{2+}$$

The binding of EPS to Fe^2+^ was studied, and it was found that the binding of Fe^2+^ increased with increasing EPS concentration. When the EPS reached a certain concentration, the Fe^2+^-EPS layer was dense on the surface of the carbon steel. Above a particular concentration, the adsorption of the Fe^2+^-EPS layer on the carbon steel surface reached dynamic equilibrium. The adsorption layer was disturbed by the absence of active adsorption sites on the surface. However, EPS binds Fe^3+^ in the solution, accelerating the dissolution of anode Fe and destroying the density of the protective layer, thus reducing corrosion inhibition. Wang et al. [65] studied the corrosion inhibition of carbon steel by Nandina domestica Thunb. extract (NDTE) in hydrochloric acid solution. The cathodic reaction mechanism was:

(6) $$2H^{+}+2e^{-}=H_{2}↑$$

The anodic reaction mechanism without NDTE [66,67] was:

(7) $$\mathrm{Fe}+{\mathrm{Cl}}^{-}↔{\left({\mathrm{FeCl}}^{-}\right)}_{\mathrm{ads}}$$

(8) $${\left({\mathrm{FeCl}}^{-}\right)}_{\mathrm{ads}}↔{\left(\mathrm{FeCl}\right)}_{\mathrm{ads}}+e^{-}$$

(9) $${\left(\mathrm{FeCl}\right)}_{\mathrm{ads}}\to {\mathrm{FeCl}}^{+}+e^{-}$$

(10) $${\mathrm{FeCl}}^{+}\to {\mathrm{Fe}}^{2+}+{\mathrm{Cl}}^{-}$$

The additional steps in the anodic reaction mechanism with NDTE [66,68] were:

(11) $${\left({\mathrm{FeCl}}^{-}\right)}_{\mathrm{ads}}+{\mathrm{InhH}}^{+}↔{\left({\mathrm{FeCl}}^{-}{\mathrm{InhH}}^{+}\right)}_{\mathrm{ads}}$$

(12) $${\left({\mathrm{FeCl}}^{-}\right)}_{\mathrm{ads}}+{\mathrm{InhH}}^{+}↔{\left({\mathrm{FeInhH}}^{+}\right)}_{\mathrm{ads}}+{\mathrm{Cl}}^{-}$$

A variety of biomass corrosion inhibitors have been found, mainly for substances containing C=O, C=N, N=N, –OH, –SH, and other functional groups, and heterocyclic organic compounds containing N, O, S, P, and other heteroatoms, which are adsorbed on the metal surface to slow down corrosion through double bonds, π-bonds of benzene rings, and lone pairs of electrons of heteroatoms interacting with the empty d orbitals of metals.

### 4.3. Film-Forming Adsorption Type

The classification of corrosion inhibitors varies, among which they can be classified into oxide film type, adsorption film type, and precipitation film type according to the different protective films formed [69]. The oxide film is a protective film that prevents corrosion by oxidizing the metal surface when corrosion-inhibiting molecules come into contact with it. The adsorption film corrosion inhibitor in the corrosive medium on the metal surface has good adsorption, and such adsorption changes the nature of the metal surface, and inhibits the corrosion of the metal. Precipitation film refers to corrosion inhibiting molecules and ions in the water due to corrosion phenomena, thus there is a complexation reaction with metal ions in the water. The reaction forms a complex that is insoluble, thereby depositing on the metal surface and playing a protective role.

**Figure 4:**
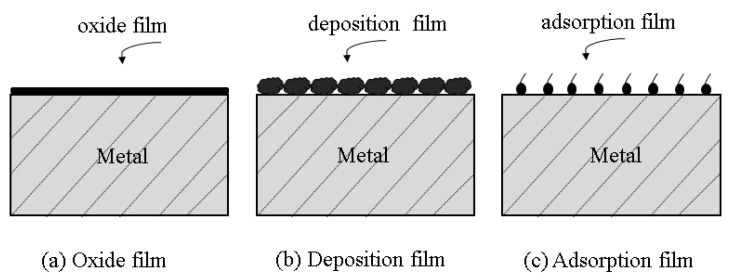
Adsorption diagram of (**a**) oxide film, (**b**) deposition film, and (**c**) adsorption film.

Stern [70] clearly defined that oxide-film-type corrosion inhibitors form a protective film containing γ-Fe_2_O_3_ on the metal surface that can extend to other adjacent locations and achieve metal protection. This protection can be based both on their oxidizing properties, through the oxidation reaction, and the formation of oxide film, thereby achieving the security of the metal. Alternatively, the corrosion inhibitor can be nonoxidizing, and can be adsorbed in the anode area with the help of dissolved oxygen in the solution to form an oxide film. Shamnamol et al. [71] illustrated that most adsorbed-film-type corrosion inhibitors are organic, mainly based on the principle of charge adsorption. Adsorption affects the surface charge while maintaining the energy unchanged, hence increasing the activation energy required for the corrosion process and decreasing the corrosion rate. Corrosion inhibitors block the metal surface and corrosion solution to prevent the occurrence of electrochemical reactions, and play a role in protecting the metal. Zhu et al. [72] explained the adsorption mechanism of precipitation-film-type corrosion inhibitors. The reaction between corrosion-inhibiting molecules and corrosive media and interaction with metal ions forms deposits on the metal surface that are difficult to dissolve in water. The film layer is thicker than the passivation film. However, its loose and easy-to-detach nature makes the protection ability poor, and the film formation effect is shown in Figure 4.

As mentioned above, the protective film separates the metal from the corrosive environment and effectively reduces the corrosion of the metal. The adsorption of biomass corrosion inhibitors is also affected by temperature, corrosive environment, electronic structure, solubility, and metal properties.

## 5. Evaluation Methods for Green Biomass Corrosion Inhibitors

The main research methods regarding the corrosion inhibition of metals by green biomass corrosion inhibitors include weight loss measurement, electrochemical analysis, surface analysis methods, inhibition mechanism analysis, and theoretical calculation studies realized through software simulations. Applying appropriate corrosion inhibition research methods to different types of extracts allows in-depth studies of their corrosion inhibition efficiency, analysis of various extract advantages and disadvantages, effects and mechanisms of action, and realization of more comprehensive characterization and analysis.

### 5.1. Weight Loss Measurement

Currently, the most commonly used approach for evaluating the efficacy of corrosion inhibitors is the measurement of weight loss. The weight loss measurement calculates the average corrosion rate of a metal sample without and with a corrosion inhibitor added [73]. Conversely, the corrosion inhibition performance of the corrosion inhibitor can be judged. The method has the advantages of simple operation, reliable results, good reproducibility, and so on. However, there are also certain disadvantages: the processing of the specimen is more complex, and for the corrosion rate of the smaller systems the experimental period is longer.

Bui et al. [74] studied the corrosion inhibiting properties of Vietnam orange peel essential oil (OPEO) in a 1 N HCl solution. Using weight loss data, the corrosion rate of mild steel was calculated. Several immersion durations were used on the steel samples. The corrosion rate (W) and inhibition efficiency (H_W_) were determined using their pre-corrosion and post-corrosion weights, as follows:

(13) $$W=\frac{m_{1}-m_{2}}{\mathrm{st}}\left({\mathrm{mg}\cdot \mathrm{cm}}^{-2}\cdot h^{-1}\right)$$

(14) $$H_{w}=\frac{w^{0}-w}{w^{0}}\times 100\%$$

where S is the surface area (cm^2^) of the sample, m_1_ and m_2_ are the sample weights before and after corrosion (mg), t is the immersion time (h), and W^0^ and W are the corrosion rates of mild steel in solutions with and without inhibitors (mg·cm^−2^·h^−1^), respectively.

Aejitha et al. [75] investigated the corrosion inhibition pattern of extracts of Commiphora caudata by weight loss measurement. The results showed that the extracts had an excellent corrosion inhibition effect on low carbon steel in 1 mol/L H_2_SO_4_ solution. The corrosion inhibition efficiency increased with increasing corrosion inhibitor mass concentration. Furthermore, the corrosion inhibition efficiency increased with increasing temperature and time, making it an effective mixed type of corrosion inhibitor. Sangeetha et al. [76] studied the corrosion inhibition of copper in nitric acid solution by Azadirachta extract. The results showed that the corrosion inhibition efficiency of Azadirachta extract increased with increasing concentrations of corrosion inhibitor, with the highest inhibition efficiency of 95.69%.

### 5.2. Electrochemical Methods

Electrochemical methods are an important tool to study the adsorption behavior of corrosion inhibitor molecules on metal surfaces. They have the characteristics of fast detection speed and simple installation of the test device. The most often utilized electrochemical measures are the potentiodynamic polarization method (Tafel) and electrochemical impedance spectroscopy (EIS). The most significant advantage of electrochemical methods over weight loss methods is the possibility of exploring more information related to corrosion inhibition mechanisms.

Electrochemical methods are usually carried out in a three-electrode system, including a working electrode, a reference electrode, and a counter electrode [77]. Various effects on the corrosion inhibition effect have been explored by adjusting the extract content, test temperature, and liquid flow rate. Electrochemical analysis is convenient, fast, and easy to use [78]. Potentiodynamic polarization can provide a variety of electrochemical parameters, such as corrosion potential (E_corr_), corrosion current density (i_corr_), anode slope (β_a_), and cathode slope (β_c_). Electrochemical impedance spectroscopy can accurately derive the corrosion rate with less influence on the electrode. Various electrochemical methods are available to evaluate the process and extent of metal corrosion, and the effectiveness and mechanism of action of corrosion inhibitors to some degree.

#### 5.2.1. Potentiodynamic Polarization Technique (Tafel)

**Figure 5:**
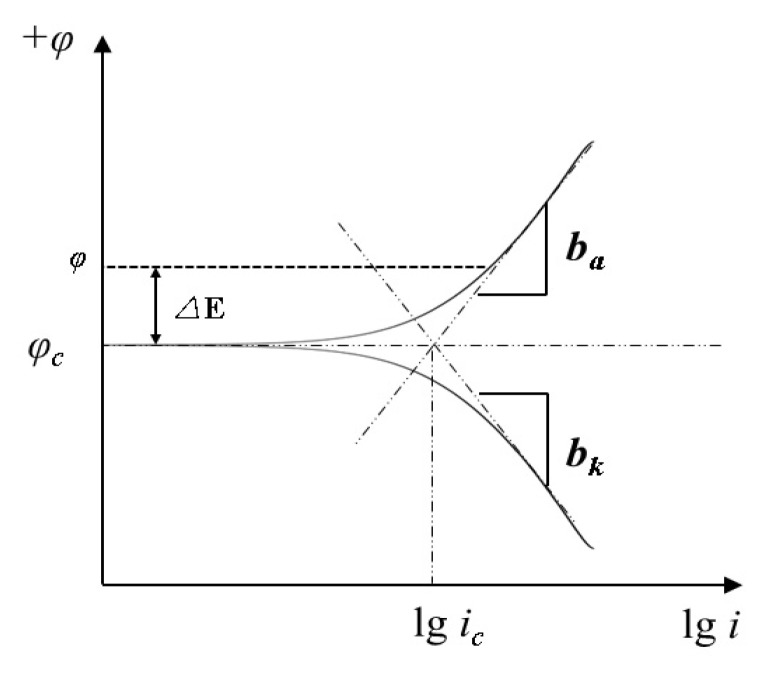
Schematic diagram of the potentiodynamic polarization curve.

The potentiodynamic polarization method is a commonly used method, and it is composed of the polarization current density and electrode potential [79]. Anodic and cathodic polarization curves comprise the relationship curves between the polarization current density and electrode potential. Nevertheless, when the potentiodynamic polarization of the potentiodynamic polarization curve method is too immense, serious curve deviations occur, and a standard potentiodynamic polarization curve cannot be obtained. Figure 5 shows a schematic of the potentiodynamic polarization curve.

Zhang et al. [80] synthesized two novel imidazole derivatives and found that they had good corrosion inhibition on low carbon steel in 1 M HCl. The conclusions of their potentiodynamic polarization curves indicated that the addition of imidazoline derivatives reduced the corrosion rate. Both the anode and cathode Tafel curves moved toward the direction of lower current density, which indicated that the coating of corrosion inhibitor molecules on the anode metal surface inhibited anode dissolution. The halogen substitution inhibitor decreased the dissolution effect of mild steel, which was positively charged when immersed in both blank solution and corrosion medium containing corrosion inhibitor. With the adsorption of chloride ions, imidazole derivative molecules formed a protective film on the steel surface, thus blocking corrosion particles. Rodrigues et al. [81] synthesized high-protein Spirulina maxima to mitigate the corrosion of carbon steel in 1 M HCl. The typical potentiodynamic polarization curve method was used in this study. According to the change in βc and βa parameters, the hydrogen evolution reaction of the cathode and metal dissolution reaction of the anode did not change with increasing corrosion inhibitor concentration. By judging the maximum displacement of OCP and E_corr_, it was concluded that Spirulina maxima is a mixed corrosion inhibitor.

Khiati et al. [82] investigated the corrosion inhibition of copper by a novel triazole derivative, bis-(4-amino-5-mercapto-1,2,4-triazol-3-yl)-butane (BAMTB), in a seawater environment using a potentiodynamic polarization curve combined with surface morphology. The results showed that the anode current density and cathode current density decreases in the presence of BAMTB, which is a mixed corrosion inhibitor. Its ability to inhibit metal corrosion is via the conventional corrosion inhibitor benzotriazole. Njoku et al. [83] extracted tobacco leaves and used a potentiodynamic polarization curve to explore their corrosion inhibition performance. They found that the results of electrochemical impedance spectra and potentiodynamic polarization curves were consistent. The experimental results showed its active dissolution character with no passivation in the potential range studied, suggesting that the extract did not alter the corrosion mechanism. Nonetheless, the introduction of the section in the acid medium caused a shift of E_corr_ to negative values. It reduced the magnitude of the anodic and cathodic current densities, with more significant cathodic effects.

#### 5.2.2. Electrochemical Impedance Spectroscopy (EIS)

**Figure 6:**
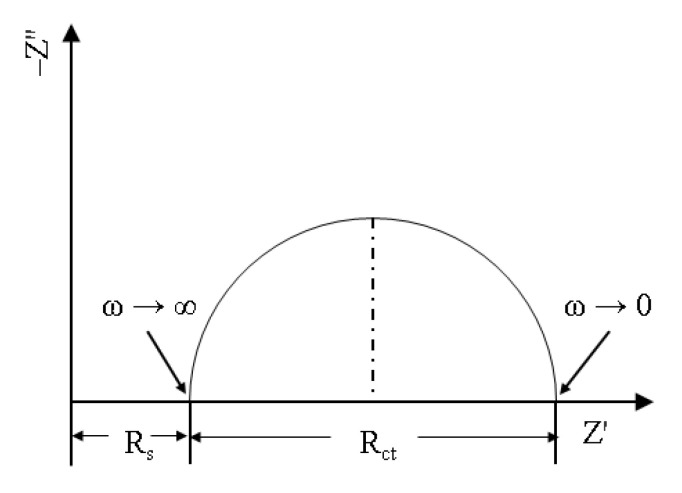
Schematic diagram of electrochemical impedance spectroscopy.

EIS is the measurement of the impedance spectra of an electrode system over a large frequency range utilizing a small-amplitude sinusoidal potential as the perturbation signal to generate an almost linear relationship between the responses of the electrode system. EIS is a common electrochemical testing technique that has a low impact on the electrode surface state. Therefore, it is very widely used in scientific research and is an important research method in electrochemical testing techniques [84]. In the equivalent circuit, Rs is the solution resistance between the working and reference electrodes, R_ct_ represents the charge transfer resistance corresponding to the corrosion reaction at the metal/electrolyte interface, and CPE is the constant phase element. In corrosion science, information on corrosion resistance, changes in electrode surface roughness, adsorption of corrosion inhibitors, and formation of corrosion products can be obtained by measuring EIS. Compared with other traditional electrochemical methods, such as potentiodynamic polarization curves, EIS provides more information on corrosion kinetics and the structure of the electrode–electrolyte solution interface, as shown in Figure 6.

Gerengi R. et al. [85] evaluated the corrosion inhibition efficiency of mimosa extract in acid rain solution on AA6060 aluminum alloy material using dynamic electrochemical impedance spectroscopy (DEIS). By adding the mimosa extract, the impedance response of AA6060 aluminum alloy in artificial acid rain solution was considerably altered. The impedance of the inhibitory effect mechanism increased as the concentration of the inhibitor increased. Using electrochemical techniques, Qiang et al. [86] studied the corrosion inhibiting characteristics of Ginkgo leaf extract (GLE) in HCl solution on X70 steel. EIS showed that the electrochemical impedance spectrum was a semicircular capacitive-resistance arc and that GLE was an effective plant-based corrosion inhibitor. Electrochemical tests demonstrated that, when the concentration of Ginkgo biloba extract increased, the corrosion current density decreased, and the effectiveness of corrosion inhibition increased.

Using EIS, Gordana et al. [87] illustrated that olive leaf extract reduced the corrosion of carbon steel in a CO_2_-saturated chloride–carbonate solution, with the efficacy of olive leaf extract rising with time. In the Nyquist graph, a semicircle in each curve represents a time constant due to the charge transfer resistance. In addition, these capacitive loops have a nonstandard shape, which originates from frequency dispersion caused by the inhomogeneity and roughness of the steel surface. Patel et al. [88] explored the corrosion inhibition efficacy of Bauhinia purpurea leaf extract in 1 N H_2_SO_4_ solution on mild steel based on conventional weight loss, electrolysis, and electrochemical impedance. Electrochemical impedance spectroscopy revealed that the primary characteristics of impedance, radiation leap resistor, and double-layer capacitor changed, mostly owing to the adsorption of active molecules, resulting in the formation of a protective layer on the surface of mild steel. Scanning electron microscopy provided conclusive evidence that the surface condition of the anticorrosive coating was improved because of the adsorption effect.

### 5.3. Metal Surface Analysis Technology

Surface analysis is performed by SEM, AFM, XPS, and FTIR. SEM and AFM analyze the metal surface shape of the corrosion inhibitor. XPS studies provide information on the elemental composition, content and chemical state of various corrosion inhibitors. To further clarify the corrosion inhibition process, energy dispersive X-ray spectroscopy and FTIR variation spectroscopy are utilized to describe the functional groups and elemental distribution of the corrosion inhibitor on the metal surface [89].

Rodrigues et al. [81] studied the microalgae Spirulina maximum biomass as a carbon steel corrosion inhibitor in 1 mol/L HCl by synthesizing high levels of natural protein inhibitors. SEM and surface morphology analysis can be used to observe the microalgae S. maxima on the surface roughness of 1020 carbon steel in 1 mol/L HCl medium. In the presence of 100 mg/L inhibitor, the surface was more uniform and rougher. The surface study indicated that microalgae in a 1 mol/L HCl solution had a significant protective effect on the 1020 carbon steel surface. Chaubey et al. [90] investigated the corrosion inhibition effect of bark extract in 1 mol/L NaOH solution on aluminum alloy. Electron microscopy revealed that adding bark extract reduced the number of pits and holes due to corrosion on the aluminum alloy’s surface. Then, 3D AFM images measured the surface roughness of the aluminum alloy. The surface roughness of aluminum alloy specimens without corrosion inhibitor addition was found to be higher, and the surface roughness decreased after addition. The results demonstrated that the bark extract had excellent corrosion resistance to aluminum alloys in alkaline environments. Chung et al. [91] examined the inhibition effect of H. fulva extract in a 1 M sulfate solution on aluminum by evaluating weight loss at several concentrations and temperatures. Energy-dispersion spectroscopy (EDX) studies revealed that the fluorescent algae extract contained Al, O, C, and N atoms present in the aluminum-alloy-adsorbed film on the specimen’s surface. AFM revealed that the H. fulva extract reduced the average surface roughness of the aluminum alloy in the corrosive solution as well. The SEM results indicated that the surface of aluminum specimens with the addition of H. fulva extract was smoother and more complete than those without the extract after soaking with sulfuric acid solution. The organic components of the extract adsorbed on the aluminum surface and inhibited the corrosion effect.

The adsorption of corrosion inhibitors on metal surfaces can be accurately characterized by several surface analysis methods. It was found that most of the extracts reduced the pores and depressions on the metal surface and decreased the average roughness. As a result of the extraction, peaks in the organic composition were evident on the metal surface, demonstrating the adsorption of corrosion inhibitors on the metal surface and the formation of protective films.

### 5.4. Computer Simulation Study

With the development of computer technology, the mechanism of action and adsorption of corrosion inhibitor molecules on metal surfaces can be explained by quantum chemical methods and molecular dynamics. Quantum chemical calculations are the computational processing of information about molecules through molecular simulation techniques using efficient computer technology to obtain information about the stable conformation of molecules and electron distribution. The interaction between corrosion inhibitor molecules and metal substrates has been investigated using molecular dynamics simulation (MDS) to obtain the stable adsorption conformation. MDS can also study the binding energy between the corrosion inhibitor molecules and the metal substrate, and the adsorption behavior of the molecules in the corrosion medium [92].

Based on molecular dynamics simulations, it has been found that the activated components all adsorb to the metal surface in parallel. In a hydrochloric acid solution, Aoufir et al. [93] investigated the corrosion inhibition mechanisms of benzimidazole derivatives (DBI). They found that the main components of the extract were imidazole derivatives, polyols, and organic acids. Their optimal conformation and front orbital distribution favored their biased adsorption on the surface of carbon steel and retarded its corrosion. Guo et al. [94] investigated the corrosion protection effects of locust bean gum (LBG) extracts on Q235 steel in a 0.5 mol/L H_2_SO_4_ solution. They found that the main components of the extract were distributed in several active sites, including nitrogen, oxygen atoms, and benzene rings, forming barrier films to the Fe and solution interfaces. Using a computer, the theoretical calculation approach may indicate that the active components of the extract form chemical bonds with the metal and generate a dense adsorption film to prevent the metal from corroding, which is a rapid and effective analytical method.

As science and technology continue to develop, quantum chemical calculations and molecular dynamics simulations as characterization methods of corrosion inhibitors will be derived from various characterization methods in the future. With the above conclusions, these characterization methods can be used to identify the composition, inhibition efficiency, and mechanism of action of corrosion inhibitors, which are practical tools for future corrosion inhibitor applications.

## 6. Conclusions

Biomass corrosion inhibitors are expected to be an alternative to traditional organic and inorganic corrosion inhibitors. Biomass corrosion inhibitors are cost-effective, accessible, biodegradable, and environmentally friendly. They can be corrosion inhibitors in mild steel, aluminum, and other metals in acid, chloride, or acid gas media. Many common weight loss techniques, electrochemistry, surface analysis, adsorption models, and other experimental and theoretical studies have revealed the promising potential of biomass as corrosion inhibitors.

In the future, the search will continue for better biomass corrosion inhibitors, more accurate characterization tools to analyze and characterize the active substances in plant, animal, and microbial extracts and to enable rapid chemical separation and extraction. The interaction of extracts with ions that typically act synergistically, such as iodide and zinc ions, and their mechanisms in the corrosion inhibition process need further investigation. In summary, with the proposed global decarbonization, research on biomass corrosion inhibitors has become a brand new direction to continue exploring the advantages of green extracts, and how to develop and utilize biomass corrosion inhibitors efficiently is a future trend of society. The future research direction of corrosion inhibitors will be low-cost, environmentally friendly, and widely applicable.

## Figures and Tables

**Table 1:** The corrosion inhibition performance of different types of corrosion inhibitors in different corrosion media.

Name	Metal	Corrosive Medium	Optimum η (%)	Ref.
**Plant**				
Ginger root	Carbon steel	HCl	95.00	[29]
Wild jute tree	Mild steel	HCl	96.90	[30]
Bitter gourd fruits	Mild steel	HCl	96.00	[31]
Binda rind	Mild steel	HCl	97.33	[32]
Betel leaves extracts	Carbon steel	HCl	94.90	[33]
Platanus acerifolia leaf	Carbon steel	NaCl	99.86	[34]
Catharanthus roseus	Mild steel	NaCl	84.00	[35]
Armoracia rusticana root	Mild steel	H_2_SO_4_	95.74	[36]
Asparagus racemosus fruits	Mild steel	H_2_SO_4_	93.25	[37]
Myristica fragrans fruit	Mild steel	H_2_SO_4_	87.81	[38]
Mutiti leaf	Low-carbon steel	H_2_SO_4_	86.23	[39]
Coreopsis tinctoria plant	Mild steel	H_2_SO_4_	80.62	[40]
**Animal**				
Phenylalanine	Steel	HCl	74.80	[41]
Glutamic acid	Aluminum	HCl	81.50	[42]
Glutamic acid	Fe-19Cr stainless steel	HCl	68.36	[43]
Polyaspartic acid–glycine	Carbon steel	NaCl	83.80	[44]
Tryptophan	Low-carbon steel	HCl	91.00	[45]
Tryptophan	Low-alloy steel	Citric acid	88.32	[46]
L-tryptophan	Low-carbon steel	HCl	92.70	[45]
Cysteine	Carbon steel	H_3_PO_4_	93.00	[47]
**Microorganisms**				
Glycolipid	Carbon steel	HCl	87.00	[26]
Marine bacterium	X80 steel	NaCl	91.16	[48]
Bacillus subtilis	Aluminum 2024		90.00	[49]
Sodium N-dodecyl asparagine (AsS) and sodium N-dodecyl arginine (ArS)	Mild steel alloy	NaCl	90.00	[50]
Ethane-1,2-diylbis(N,Ndimethyl-N-hexadecylammoniu-macetoxy)dichloride	Mild steel	HCl	98.00	[28]

## Data Availability

Not applicable.
